# Brazilian Oral Health Policy: metasynthesis of studies on the Oral Health Network

**DOI:** 10.11606/s1518-8787.2021055003454

**Published:** 2021-12-02

**Authors:** Sylvio da Costa, Pierre Guedes Araujo, Karla Frichembruder, Fernando Neves Hugo

**Affiliations:** I Secretaria Municipal Saúde Florianópolis SC Brasil Secretaria Municipal Saúde. Florianópolis, SC, Brasil; II Universidade Federal do Rio Grande do Sul Faculdade de Odontologia Centro de Pesquisas em Odontologia Social Porto Alegre RS Brasil Universidade Federal do Rio Grande do Sul. Faculdade de Odontologia. Centro de Pesquisas em Odontologia Social. Porto Alegre, RS, Brasil; III Universidade Federal do Rio Grande do Sul Faculdade de Odontologia Departamento de Odontologia Preventiva e Social Porto Alegre RS Brasil Universidade Federal do Rio Grande do Sul. Faculdade de Odontologia. Departamento de Odontologia Preventiva e Social. Porto Alegre, RS, Brasil

**Keywords:** Dental Health Services, Health Care Quality, Access, and Evaluation, Unified Health System, Systematic Review

## Abstract

**OBJECTIVE:**

Analyze the performance of the oriented oral health care network from its implementation, in 2004 to 2020, according to publications on the subject.

**METHODS:**

This is a research with a methodological description of metasynthesis.

**RESULTS:**

The searches resulted in 600 complete publications (586 in the first search and another 14 in the second search), according to the established criteria. 539 articles were excluded: 151 after duplication analysis, 236 after reading the title, 45 by type of publication and 107 after reading the abstract, as they did not fit the research theme. Thus, 61 original publications and another 29 publications in snowball sampling were selected and analyzed, totaling 90 publications. From this selection, we chose to use the model proposed by the *Projeto de Avaliações de Desempenho de Sistemas de Saúde* (PROADESS — Health Systems Performance Assessment Project). The study will use its guiding principles on the dimension “Health Services Performance”.

**CONCLUSION:**

The analyzed set allowed us to identify that the way the Brazilian State organizes and finances oral health care made it possible to expand access and the number of procedures performed, but not the creation of an effective comprehensive care network, after more than a decade of implementation of *Brasil Sorridente* (Smiling Brazil).

## INTRODUCTION

The Brazilian Oral Health Policy (PNSB) was built in a context of political-institutional transformations in Brazil during the 1980s and 1990s, from the end of the military dictatorship and the beginning of the so-called New Republic. In this set of changes in the country’s political regime, the foundations of the Brazilian Unified Health System (SUS) are laid, more specifically in articles no. 196 to 200 of the 1988 Federal Constitution. Considering the entire legal system and legal framework implemented from the 1988 Constitution to the implementation of SUS, dentistry was present in a peripheral way until 2003^1,2^. The Ministerial Ordinance no. 1,444, which includes dentistry in the Family Health Program (PSF), took place more than five years after the program’s implementation, with a clear prejudice for the oral health inclusion in the health teams^3^.

Even after the implementation of the aforementioned ordinance, the real insertion of oral health in SUS only happened after 2003, with the Smiling Brazil program, which, through a different legal system, provided a rapid increase in resources for the expansion of services and creation of public facilities that did not exist until then, enabling the conditions for the formation of a comprehensive network of oral health care^1^.

The justification for the study is that the expansion of public facilities in oral health may not have created a comprehensive care network with varying levels of care, but rather a robust set of atomized public facilities, without integration and producing inequities, a phenomenon described in the literature as the “inverse care law”^4^. Thus, the purpose of the study is to analyze the oral health care network performance, based on the implementation of PNSB.

## METHODS

This is a research with a methodological description of metasynthesis, placing itself as a multifocal study of a phenomenon. Thus, searches were conducted for articles published between 2004 and 2020 on the subject. This study is the result of the doctoral thesis of one of the authors, on the Brazilian Oral Health Policy, developed in the Postgraduate Program in Dentistry at the Universidade Federal do Rio Grande do Sul (UFRGS).

### Search Strategy

The article search strategy for the research used keywords/free terms as follows: ((oral health OR dentistry) AND (public policy OR oral health services) OR (Smiling Brazil)). These terms were used to ensure that a greater number of titles and abstracts were obtained, resulting in a wide-ranging search. Thus, the search happened in February and March 2021, in the following electronic databases: Scientific Electronic Library Online (SciELO), Latin American and Caribbean Literature in Health Sciences (Lilacs) and Medical Literature, Analysis and Retrieval System Online (Medline). A search was made with keywords in Portuguese and English in the databases mentioned above. Due to the characteristics of the PubMed database, the search engine used was as follows: (“oral health” [All Fields] OR “dentistry” [All Fields]) AND (“public policy” [All Fields] OR “oral health services” [All Fields]) OR (Brazil [All Fields] AND smiling [All Fields]) AND ((pubmed books [filter] OR Classical Article [ptyp] OR Clinical Conference [ptyp] OR Comparative Study [ptyp] OR Evaluation Studies [ptyp] OR Historical Article [ptyp] OR Review [ptyp] OR Scientific Integrity Review [ptyp] OR Interview [ptyp] OR Meta-Analysis [ptyp] OR Newspaper Article [ptyp] OR Observational Study [ptyp] OR Overall [ptyp] OR Personal Narratives [ptyp]) AND (“2004/01/01” [PDAT]: “2020/12/31” [PDAT]) AND “humans” [MeSHTerms]) AND “loattrfulltext” [sb].

As the study evaluates the effectiveness and performance of the PNSB, a second search was carried out, added to the first, covering articles related to the *Programa Nacional de Melhoria do Acesso e da Qualidade* (PMAQ – Brazilian Program for Access and Quality Improvement) in the oral health axis, as the program traces a large national panorama of Oral Health Networks^5^. Thus, studies based on the PMAQ were evaluated, as the organization and integration of health care networks are one of the program’s evaluations subdimensions, which is included in the teams’ evaluation tool. Therefore, the usefulness of searching for additional studies was considered. The article search strategy was similar to the first one, using keywords/free terms as follows: PMAQ OR (Program AND Improving AND Access AND Quality) AND “Brazil” AND (“service” AND “health” AND “dental”). Thus, the search happened in February and March 2021, with keywords in Portuguese and English, in the following electronic databases: Scientific Electronic Library Online (SciELO), Latin American and Caribbean Literature in Health Sciences (Lilacs) and Medical Literature, Analysis and Retrieval System Online (Medline). Due to the characteristics of the PubMed database, the search engine used was as follows: PMAQ [All Fields] OR (Program [All Fields] AND Improving [All Fields] AND Access [All Fields] AND Quality [All Fields]) AND (“brazil” [MeSHTerms] OR “brazil” [All Fields]) AND (“dental health services” [MeSHTerms] OR (“dental” [All Fields] AND “health” [All Fields] AND “services” [All Fields]) OR “dental health services” [All Fields] OR “dental”[All Fields]).

Once the searches were completed, the metasearch engine available on the journal’s portal of the *Coordenação de Aperfeiçoamento de Pessoal de Nível Superior* (Capes — Coordination for the Improvement of Higher Education Personnel) was used to find new articles not selected in the previous step, using the same keywords and filters. However, this search came back with no results.

Completing the article selection, the snowball sampling methodology was used, a type of non-probabilistic sampling that uses chains or reference networks of previously selected articles in the search. The use of this technique took place to reach the so-called saturation point, in which there would be robust content in the selection of material and a collection of sufficient information for research. From the snowball sampling, another 29 articles were selected, distributed as follows: 25 articles from the SciELO database and 4 from the Lilacs database. To choose and select these articles, the same criteria was used by two reviewers and arbitration, in case of disagreement, by a third reviewer. All selected articles are displayed in the Tables 1, 2, 3 and 4.

**Table 1:** Distribution of selected articles by journal.

Journal’s name	Quantity of selected publications
*Arquivos do Centro de Estudos da Faculdade de Odontologia da Universidade Federal de Minas Gerais*	3
Brazilian Dental Journal	3
*Cadernos de Saúde Pública*	13
*Revista Ciência & Saúde Coletiva*	21
*Epidemiologia e Serviços de Saúde*	2
*Revista de Saúde Publica*	7
*Revista da ABENO*	2
*Revista de Administração Pública*	2
*Revista de APS – Atenção Primária à Saúde*	3
*Revista Baiana de Saúde Pública*	3
*Revista Brasileira de Medicina de Família e Comunidade*	1
*Revista Brasileira em Promoção da Saúde*	1
*Revista de Odontologia da UNESP*	4
*Revista Gaúcha de Odontologia*	4
*Revista Odontológica do Brasil Central*	1
*Saúde em Debate*	8
*Saúde e Sociedade*	3
*Stomatos*	1
*Trabalho, Educação e Saúde*	3
*Revista Pesquisa Brasileira em Odontopediatria e Clínica Integrada*	2
Physis: *Revista de Saúde Coletiva*	2
*Revista Ciência Plural*	1
Overall total	90

**Table 2:** Distribution of selected articles by year of publication.

Year of publication	Title count
2005	4
2006	2
2007	4
2008	5
2009	7
2010	8
2011	3
2012	9
2013	7
2014	4
2015	7
2016	4
2017	3
2018	13
2019	8
2020	2
Overall total	90

**Table 3:** Distribution of selected articles by study design.

Study design	Title count
Quasi-randomized community trial	1
Economic evaluation study	1
Before-and-after intervention study	1
Descriptive study based on health service professionals	2
Descriptive study based on health services	2
Descriptive study based on health service users	1
Prevalence descriptive study based on health professionals	4
Prevalence descriptive study based on health services	1
Ecological study	3
Ecological time-series study	6
Ecological cross-sectional study	1
Longitudinal study based on health services	1
Longitudinal study based on health service users	1
Qualitative study	35
Quantitative and qualitative study	12
Cross-sectional study	1
Cross-sectional study based on health professionals	1
Cross-sectional study based on health service professionals	3
Cross-sectional study based on health services	6
Cross-sectional study based on health service users	7
Overall total	90

**Table 4:** Distribution of selected articles by health services performance evaluation sub-dimension.

Evaluation sub-dimension	n
Accessibility	13
Continuity	44
Effectiveness	33
Overall total	90

For the article selection, the following inclusion criteria were considered: a) original articles in Portuguese, Spanish and English, published in indexed journals; b) articles published in the period between 2004 and 2018, as the PNSB, under the name Smiling Brazil, was implemented in 2004, and therefore it is understood that the study on the policy evolution should be carried out in the period established above. Only articles with available full text were considered.

### Methodological Approach

We chose to use the evaluation model proposed in the *Projeto de Avaliações de Desempenho de Sistemas de Saúde* (PROADESS — Health Systems Performance Assessment Project), which constitutes one of the most comprehensive and structured proposals for evaluating the health systems performance, with a matrix that incorporates important dimensions critical for the Brazilian Unified Health System (SUS) evaluation. The PROADESS Conceptual Matrix^6^ brings four major dimensions for the evaluation of the Health System: determinants of health, health conditions of the population, health system and performance of health services, since the equity axis cuts across all these dimensions and performance must include the sub-dimensions access, effectiveness, efficacy, adequacy, continuity, safety, acceptability, and patients’ rights.

## RESULTS

The established criteria resulted in 600 complete publications (586 in the first search and 14 in the second). 539 articles were excluded: 151 after duplication analysis, 236 after reading the title, 45 by type of publication and 107 after reading the abstract, as they did not fit the research theme. In the end, 61 original publications and another 29 publications in snowball sampling were selected and analyzed, totaling 90 publications.

The selection of articles was performed by two different reviewers (R1 and R2) who, based on the constructed search key, individually examined the articles, following the same eligibility criteria, such as title reading, publication type and reading of the abstract for further definition of its relevance to the scope of this study, as shown in Flowchart 1. Thereafter, the reviewers applied eligibility criteria alone in the full reading of the selected articles, crossing information to certify the integrity of the content, considering a set of criteria, such as year of publication, study design, sample characteristics (sample size, study location and period considered in the study), among others. In case of doubt, a third reviewer (R3) was consulted, when there were disagreements, debates were held until an agreement for the inclusion or exclusion of the article was reached.

To analyze the articles and better understand the configuration of the implantation and implementation of the health care network, the studies were categorized into thematic axes: 1) access, 2) continuity and 3) effectiveness. In this process, an important gap was observed, as productions classified in the other five dimensions considered by the PROADESS Conceptual Matrix for performance evaluation (safety, adequacy, acceptability, efficiency, and respect for the law) were not found.

The basic concepts of access, continuity and effectiveness can be defined, respectively, as the user’s ability to use health services and assistance at the appropriate time, the competence of the health network to provide care in a continuous and orderly manner and, finally, achieving the best possible result in the patient’s health care.

## DISCUSSION

### Access

Several national studies^7^ focused on the provision of oral health services based on access, its inequalities and the form of use in a care network. In Peres’ work^8^ on the use of services in SUS, the search for oral health care was the third largest demand.

In the field of primary care, the PNSB has unequivocally constituted an important continuity tool, guided by fundamental principles of integrality, universality and equity, in which the existence of one of these principles cannot be understood without the inclusion of the others. To achieve these principles, it is necessary to comply with local planning stages, including situational diagnosis, demand prioritization, scheduling for the execution of actions, execution of assistance, monitoring and evaluation. However, all legal order of the Brazilian Oral Health Policy bypasses the organization of the provision of services and the coordination of the assistance network, mainly focused on the installation of public oral health facilities. Municipalities have experimented with the most diverse forms of organizing the provision of oral health services in primary care, to the extent that the reception of users is given by free demand, by programmed actions, by urgent care or even by combinations of these, without a defined minimum standard of quality^9^ or the presence of health access indicators that have been the object of local-regional planning for the network formation.

The studies^10,11^ identified an overload in the emergency care of low clinical complexity in emergency care units, which could have been assessed in primary care, but ended up transforming emergency care into the health system’s fundamental gateway, disorganizing the network. In a national study^8^, it was observed that, between 1998 and 2008, the great expansion in the access to oral health services was evidenced among young people aged 7 to 19 years old, thus reporting to the old model of dental services aimed at schoolchildren. Figueiredo and Góes^12^ revealed important inequalities in the access to oral health services but recognized the increase in coverage due to the reduction in the number of people who have never been to the dentist. Silva^13^ showed that high coverage of Oral Health teams is easily found in municipalities with a small population, where with a small number of teams is easy to achieve a coverage close to 100% of the population. However, inadequate models of supply, coverage and public spending on health still remain^14^, as the model gives the local manager the freedom to organize municipal networks and does not make clear the organization and financing of regionalized health care networks, nor does it point to the common figure in countries with universal health networks of a regional health authority that would organize this network.

Casotti^15^, in a study on the results of the first cycle of the PMAQ-AB, pointed out that simply making an appointment is not accessible to 34.5% of users interviewed in more than 12 evaluated oral health teams. The study also identified that 33% of users who sought care were treated on the same day (spontaneous demand and/or emergency cases) and that 45.8% of patients were scheduled within 2 to 15 days after making the appointment (programmed demand). There were significant macro-regional differences, with 48.5% in the North Region being treated on the same day or the following day and 39.7% being scheduled within 2 to 15 hours after making the appointment; and in the Southeast Region 19.2% were treated on the same day or the following day, 19.1% between 16 and 30 days after making the appointment and 14% waited more than 30 days after making the appointment.

Greater coverage of primary care and greater access to services or resoluteness are not necessarily related, as several studies have shown^16,17^, because the opening of a health service must be guided by the epidemiological need of the territory, following a plan guided by a set of indicators that monitor and evaluate the health network performance^7^, in other words, did the increase in access make it possible for a certain time to improve the population’s health indicators? Or, has the opening of a service made it possible for those who most need this service to be able to access it in a timely manner? Other articles^8,10^ point to a scarcity of studies on the monitoring of indicators and the evaluation of the quality of services provided, in which, for example, Peres^8^ points to an imbalance between the use of health services and social groups, with evident damage to the economically most fragile.

In a study carried out^9^ in the metropolitan region of Curitiba, it was found that, in the Oral Health Care Network, access is insufficient and unequal, according to the author, due to the poor organization in the planning of this line of care and assistance interventions with still strong influence of former school health programs.

### Effectiveness

Effectiveness is defined as the achievement of established goals, that is, the improvement of oral health indicators and the improvement of the population’s oral health condition within the service network at the levels of care, seeking comprehensiveness. In another perspective, the population expects to have their oral health demands resolved by the service^18^, and the search for effectiveness involves the integration of services in care networks, which should aim at the efficiency and rationality of services, producing savings, expanding assistance, improving access and avoiding duplication of service^13^.

Casotti^15^, analyzing the data from the first cycle of the PMAQ, found that almost half of the oral health teams in Brazil do not have any protocol guiding the management of the clinic or the organization of its work processes, which indicates little rationality in the routine of health assistance by the teams. Different results were found in the Dental Specialties Centers (CEO), in which 78.2% of dentists reported having defined reference and counter-reference flows in their work routines. In the same study, data from the first cycle of the PMAQ show that 85.1% of the oral health teams have spontaneous demand, but only 43.8% use some protocol for it, which corroborates the other results found in several studies^18^.

When we analyze the waiting time for a reference appointment in the CEO, we realize that stomatology is the specialty with the longest waiting time, with 48.3% of all patients waiting more than 1 year for an appointment, reaching 63.8% in the North Region and 56.5% in the Northeast Region, indicating the fragility of an unprepared and unresolvable network to receive this patient with suspected cancer.

Studies on the CEO conducted nationally and specifically in the Southern Region of Brazil^21,22^ found that most CEO do not meet the monthly production goals stipulated in the specific Ordinance for receiving federal funding resources. Most of the goals achieved were those of basic procedures, making the medium complexity service compete with the primary care service, corroborating the previous argument, raised by Chaves^19^, Soares^23^ and Tomassi^24^, and putting medium complexity, in practice, as another gateway to the system and setting up a dual-gateway network. Also, according to Moura^22^, this result can also indicate low resolution of primary care, a perspective corroborated by Barbosa^25^, who analyzed the profile of referrals to medium complexity through the regulation system, in which a considerable range of referrals to other points of care in the network were procedures of low technological complexity, which could be carried out in primary care, without the need of physical displacement of the SUS user, delays on solving cases and other disorders resulting from the worsening of the disease patterns due to waiting.

### Continuity

In a care network, the different points of care must be interconnected, forming communicating vessels where a change in one point, directly or indirectly, interferes with the others. Thus, when there is a notable change in some health indicator at one point, the others also reflect this change in pattern. Several studies indicate the existence of isolated health services, without major relations with other care points, as pointed out by Scarparo^20^, that even with the increase in the indicator of first programmatic dental appointments and extractions in primary care, the analyzed network coexists with indicators of endodontics and periodontics production (secondary care), constant in the analyzed period, that is, the number of people treated in primary care increased, but there was no follow-up in secondary care, similar behavior was found in Cuiabá^26^ and Rio Grande do Norte^27^, where dental indicators behaved irregularly and without complying with clear causal relationships.

As most Brazilian municipalities are small, a medium-complexity dental network that can only offer primary care services is not justified^28^, this reality breaks the logic of continued treatment within the same municipality, consequently requiring inter-municipal and state covenants. Thus, the continuity of care and the comprehensiveness of care offered by SUS depend on multilateral agreements between different social and political factors, which makes its implementation difficult.

In a study^20^ encompassing all municipalities in the State of Rio de Janeiro, between 2004 and 2010, an increase in the first programmatic dental appointment in small and large municipalities was shown, while a retraction in medium-sized municipalities happened. An expansion in all cities of primary care procedures per inhabitant was also shown, leading to an increase in the continuity of treatment. However, in her study, Mattos^17^noted the overlapping of some treatment’s availability, lack of others and precarious integration between the services, that is, the continuity of care, a pillar of comprehensive care, in which the user should have an organized set of services available, is affected by the expansion of public facilities without planning, which directly interferes in the evolution of their morbidity degree with an impact on the outcome.

A study^28^ on the reference and counter-reference system in the metropolitan region of Rio de Janeiro observed low governance of local administrations regarding the availability of appointments in primary care for medium-complexity services, creating a dangerous funnel and a long waiting list, which generates anguish for users and makes the expectation of comprehensive care more complex. In this study, the factors that influence the formation of long waiting lists, for specialists, are similar to those observed in other studies, such as a precarious mechanism for regulating appointments and limited availability of medium-complexity services. It is assumed that those available appointments in medium complexity, with specialists, are made available without calculating the real need for absorption of these patients from primary care, as if the need for specialized treatments had to adapt to the number of available slots and not the other way around. If we compare it to the Brazilian popular saying, the offer of vacancies in medium complexity is a tailor, who, when noticing that the suit he made is smaller than the customer, decides to change the customer and not the suit.

Another component of inequality is the municipalities with better conditions, which are more likely to offer comprehensive care. Within these same municipalities, there are specific groups with better health conditions, becoming groups with more access to services. In this sense, waiting lists are symptoms of an insufficient service which does not absorb a certain demand, making the size of the waiting list for health services inversely proportional to their need, that is, long (large) lists deduct insufficient network (small), making the network itself ineffective.

Several authors^13,15,16,22^ point out that, in the set of other health policies, an expansion of the services offered without planning and without programming actions exists. Mattos^17^ considers that the vertiginous expansion of oral health services in primary care has not followed the timid expansion of the medium-complexity network, thus not guaranteeing comprehensive care for users.

Focusing on the CEO’s spatial occupancy in the five Brazilian macro-regions, Góes^21^ demonstrated that most CEOs were concentrated in the Northeast and then the Southeast Region, leading to the belief that the principle of equity was being conducted, given the great socio-economic fragility of the region. However, it is also noted in the same study that the North Region, which is also socioeconomically fragile, has the lowest concentration of CEO, even though it is not the least inhabited region, which suggests that equity was not a central axis in the distribution of these facilities.

In another study by Góes^21^, it was observed that the implemented CEOs were in more populous municipalities, with higher volumes of municipal revenue for investment, better Human Development Index (HDI) and, consequently, better oral health conditions, results corroborated by other research^30^.

Therefore, the analysis of the existing obstacles and the limitations presented for the fulfillment of its objective must not obscure the progress achieved. Clinical protocols, reference and counter-reference systems, offered services portfolios and regulation of certain actions bring greater rationality to a healthcare network. It should also be noted that, despite the significant percentage of municipalities that implemented oral health teams after the policy was implemented, this study still showed low population coverage, showing that the number of teams implemented in the municipalities is still small, requiring to be increased to treat the population. Authors such as Góes point to the need to review the specialties’ services offered by CEOs, where the greatest demands for services, such as endodontics, are expanded and specialties with lesser demands in oral health are inserted in the perspective of loco-regional planning, respecting the specificity from certain health regions.

Therefore, even with the significant increase and diversification of the care network, several signs indicate that the organization of services and their form of management hinder the formation of regionalized networks of comprehensive health care. It is also noteworthy that, although the increase in the number of health units and professionals in care has an impact on the use of the service offered, there are no institutionalized mechanisms for measuring the quality of the provided service, nor strong execution of goals and indicators, except for the three PMAQ-AB cycles.

Observing the PROADESS Conceptual Matrix and its eight sub-dimensions, another crucial point the selected articles brought was the small number of studies that address different themes of access, effectiveness and continuity, that is, the volume of publications found lacked thematic diversity. Thus, it is necessary to evaluate the Oral Health Network in a more comprehensive way and in its most varied aspects.

Despite efforts in the search for the greatest possible amount of articles on the topic addressed, an important limitation of the study is the lack of articles published in the researched sources. Although there are few articles on the subject and its variables, this does not disqualify the results found given the relevance of the selected articles, as we also do not seek to exhaust the subject, but to add it in the perspective of a better understanding of the implemented policy.
