# Supplementary material for: Brazilian Oral Health Policy: metasynthesis of studies on the Oral Health Network
Source: Rev Saude Publica. 2021 Dec 2;55:105. doi: 10.11606/s1518-8787.2021055003454 (PMC8664239; doi:10.11606/s1518-8787.2021055003454)
Supplement: Translation

# Translation: Política Nacional de Saúde Bucal: metassíntese dos estudos sobre a Rede de Saúde Bucal

**Authors:** Sylvio da Costa, Pierre Guedes Araujo, Karla Frichembruder, Fernando Neves Hugo

**Affiliations:** I Brasil Secretaria Municipal Saúde. Florianópolis, SC, Brasil; II Brasil Universidade Federal do Rio Grande do Sul. Faculdade de Odontologia. Centro de Pesquisas em Odontologia Social. Porto Alegre, RS, Brasil; III Brasil Universidade Federal do Rio Grande do Sul. Faculdade de Odontologia. Departamento de Odontologia Preventiva e Social. Porto Alegre, RS, Brasil

**Keywords:** Serviços de Saúde Bucal, Qualidade, Acesso e Avaliação da Assistência à Saúde, Sistema Único de Saúde, Revisão Sistemática

## Abstract

**OBJETIVO:**

Analisar o desempenho da rede de cuidados em saúde bucal orientada a partir sua implantação, em 2004 até 2020, segundo publicações sobre o tema.

**MÉTODOS:**

Trata-se de uma pesquisa com descrição metodológica de metassíntese.

**RESULTADOS:**

As buscas resultaram em 600 publicações completas (586 na primeira busca e mais 14 na segunda busca), de acordo com os critérios estabelecidos. Foram excluídos 539 artigos: 151 artigos após análise de duplicidade, 236 artigos após a leitura do título, 45 pelo tipo de publicação e 107 artigos após leitura do resumo, por não se enquadrarem na temática da pesquisa. Dessa forma, foram selecionadas e analisadas 61 publicações originais e mais 29 publicações em *snowball sampling*, totalizando 90 publicações. A partir dessa seleção,optou-se por usaro modelo proposto pelo Projeto de Avaliações de Desempenho de Sistemas de Saúde – PROADESS. O estudo se utilizará de seus princípios norteadores sobre a dimensão “Desempenho dos Serviços de Saúde”.

**CONCLUSÃO:**

O conjunto analisado permitiu identificar que a forma como o Estado brasileiro organiza e financia o atendimento em saúde bucal possibilitou a ampliação do acesso e do número de procedimentos realizados, mas não da criação de uma efetiva Rede de cuidados integrais, após mais de uma década de implementação do Brasil Sorridente.

## INTRODUÇÃO

A Política Nacional de Saúde Bucal (PNSB) foi construída em um contexto de transformações político-institucionais ocorridas no Brasil durante as décadas de 1980 e 1990, a partir do término da ditadura militar e início da chamada Nova República. Nesse conjunto de mudanças no regime político do país, são lançadas as bases do Sistema Único da Saúde (SUS), mais especificamente nos artigos de nº196 a 200 da Constituição Federal de 1988. Considerando todo o ordenamento legal e arcabouço jurídico implantado a partir da Constituição de 1988 para implementação do SUS, a odontologia esteve presente até 2003 de maneira periférica^1,2^. A Portaria Ministerial nº 1.444, que inclui a odontologia no Programa Saúde da Família (PSF), aconteceu mais de cinco anos depois da implantação do programa, com claro prejuízo para inserção da saúde bucal nas equipes de saúde^3^.

Mesmo após à implantação da portaria citada, a real inserção da saúde bucal no SUS só aconteceu a partir de 2003, com o programa Brasil Sorridente, que por meio de um diverso ordenamento legal-jurídico, proporcionou um vertiginoso aumento de recursos para ampliação de serviços e criação de equipamentos públicos não existentes até então, possibilitando as condições para a formação de uma rede integral de cuidados em saúde bucal^1^.

A justificativa do estudo é que a ampliação de equipamentos públicos em saúde bucal pode não ter criado uma Rede de cuidados integrada de variados níveis de atenção, mas sim um conjunto robusto de equipamentos públicos atomizados, sem integração e produtor de iniquidades, fenômeno descrito na literatura como “lei do cuidado inverso”^4^. Assim, o objetivo do estudo é analisar o desempenho da rede de cuidados em saúde bucal, a partir da implantação da PNSB.

## MÉTODOS

Trata-se de uma pesquisa com descrição metodológica de uma metassíntese, se colocando como um estudo multifocal de um fenômeno. Assim, realizou-se buscas de artigos publicados entre 2004 e 2020 sobre a temática. Este estudo é fruto da tese de doutorado de um dos autores, sobre Política Nacional de Saúde Bucal, desenvolvida no Programa de Pós-Graduação em Odontologia da Universidade Federal do Rio Grande do Sul (UFRGS).

### Estratégia de Busca

A estratégia de busca de trabalhos para a pesquisa utilizou descritores/termos livres da seguinte forma: ((saúde bucal *OR* odontologia) *AND* (política pública *OR* serviços de saúde bucal) *OR* (Brasil Sorridente)). Esses termos foram utilizados para garantir que um número maior de títulos e resumos fosse obtido, resultando em uma busca mais abrangente. Assim, a busca se deu nos meses de fevereiro e março de 2021, nas seguintes bases de dados eletrônicas: *Scientific Electronic Library Online* (SciELO), Literatura Latino-Americana e do Caribe em Ciências da Saúde (Lilacs) e *Medical Literature Analysis and Retrieval System Online* (Medline). Foi utilizada a busca com os descritores em português e inglês nas bases de dados citadas acima. Pelas características da base PubMed, o buscador utilizado foi da seguinte forma: (“*oral health*” [*All Fields*] *OR* “*dentistry*” [*All Fields*]) *AND* (“*public policy*” [*All Fields*] *OR* “*oral health services*” [*All Fields*]) *OR* (Brasil [*All Fields*] *AND* sorridente [*All Fields*]) *AND* ((pubmed *books* [*filter*] *OR Classical Article* [*ptyp*] *OR Clinical Conference* [*ptyp*] *OR Comparative Study* [*ptyp*] *OR Evaluation Studies* [*ptyp*] *OR Historical Article* [*ptyp*] *OR Review* [*ptyp*] *OR Scientific Integrity Review* [*ptyp*] O*R Interview* [*ptyp*] *OR Meta-Analysis* [*ptyp*] *OR Newspaper Article* [*ptyp*] *OR Observational Study* [*ptyp*] *OR Overall* [*ptyp*] *OR Personal Narratives* [*ptyp*]) *AND* (“2004/01/01” [*PDAT*]: “2020/12/31” [*PDAT*]) *AND* “*humans*” [*MeSHTerms*]) *AND* “*loattrfulltext*” [s*b*].

Como o estudo avalia a efetividade e desempenho da PNSB, realizou-se uma segunda busca, agregada à primeira, abarcando artigos relativos ao Programa Nacional de Melhoria do Acesso e da Qualidade (PMAQ) no eixo saúde bucal, uma vez que o programa traça um grande panorama nacional das Redes de Saúde Bucal^5^. Assim, avaliou-se estudos baseados no PMAQ, pois a organização e integração das redes de atenção à saúde é uma das subdimensões de avaliação do programa, que consta do instrumento de avaliação das equipes. Portanto, considerou-se a utilidade da busca de estudos adicionais. A estratégia de busca de trabalhos foi simular à primeira, utilizando descritores/termos livres da seguinte forma: PMAQ OR (Programa *AND* Melhoria *AND* Acesso *AND* Qualidade) *AND* “Brasil” *AND* (“serviço” *AND* “saúde” *AND* “bucal”). Assim, a busca se deu nos meses de fevereiro e março de 2021, com descritores em português e em inglês, nas seguintes bases de dados eletrônicas: *Scientific Electronic Library Online* (SciELO), Literatura Latino-Americana e do Caribe em Ciências da Saúde (Lilacs) e *Medical Literature Analysis and Retrieval System Online* (Medline). Pelas características da base PubMed, o buscador utilizado foi da seguinte forma: PMAQ [*All Fields*] *OR* (*Program [All Fields] AND Improving [All Fields] AND Access [All Fields] AND Quality [All Fields]) AND (“brazil” [MeSHTerms] OR “brazil” [All Fields]) AND (“dental health services” [MeSHTerms] OR (“dental” [All Fields] AND “health” [All Fields] AND “services” [All Fields]) OR “dental health services” [All Fields] OR “dental” [All Fields])*.

Concluídas as busas, utilizous o metabuscor disponível no portal de periódicos da Coordenação de Aperfeiçoamento de Pessoal de Nível Superior (Capes), para encontrar novos artigos não selecionados na etapa anterior, utilizando os mesmos descritores e filtros. Contudo, não houve retorno nessa busca.

Finalizando a seleção dos artigos, foi utilizada a metodologia bola de neve (*snowball sampling*), um tipo de amostragem não probabilística que emprega cadeias ou redes de referência dos artigos selecionados anteriormente na busca. A utilização dessa técnica se deu para alcançar o chamado ponto de saturação em que haveria robustez do conteúdo na seleção do material e uma coletânea de informações suficientes para pesquisa. A partir da bola de neve, selecionou-se mais 29 artigos assim distribuídos: 25 artigos da base SciELO e 4 da base Lilacs. Para escolha e seleção desses artigos, utilizou-se os mesmos critérios, por dois revisores e arbitragem, em caso de discordâncias, por um terceiro revisor. Todos os artigos selecionados estão expostos nas Tabelas 1, 2, 3 e 4.

**Tabela 1:** Distribuição dos artigos selecionados por periódico.

Nome do periódico	Quantidade de publicações selecionadas
Arquivos do Centro de Estudos da Faculdade de Odontologia da Universidade Federal de Minas Gerais	3
*Brazilian Dental Journal*	3
Cadernos de Saúde Pública	13
Revista Ciência & Saúde Coletiva	21
Epidemiologia e Serviços de Saúde	2
Revista de Saúde Publica	7
Revista da ABENO	2
Revista de Administração Pública	2
Revista de APS – Atenção Primária à Saúde	3
Revista Baiana de Saúde Pública	3
Revista Brasileira de Medicina de Família e Comunidade	1
Revista Brasileira em Promoção da Saúde	1
Revista de Odontologia da UNESP	4
Revista Gaúcha de Odontologia	4
Revista Odontológica do Brasil Central	1
Saúde em Debate	8
Saúde e Sociedade	3
Stomatos	1
Trabalho, Educação e Saúde	3
Revista Pesquisa Brasileira em Odontopediatria e Clínica Integrada	2
*Physis:* Revista de Saúde Coletiva	2
Revista Ciência Plural	1
Total geral	90

**Tabela 2:** Distribuição dos artigos selecionados, por ano de publicação.

Ano de publicação	Contagem de título
2005	4
2006	2
2007	4
2008	5
2009	7
2010	8
2011	3
2012	9
2013	7
2014	4
2015	7
2016	4
2017	3
2018	13
2019	8
2020	2
Total geral	90

**Tabela 3:** Distribuição dos artigos selecionados por delineamento

Delineamento do estudo	Contagem de título
Ensaio comunitário quase-randomizado	1
Estudo de avaliação econômica	1
Estudo de intervenção do tipo antes-depois	1
Estudo descritivo com base em profissionais de serviços de saúde	2
Estudo descritivo com base em serviços de saúde	2
Estudo descritivo com base em usuários de serviços de saúde	1
Estudo descritivo de prevalência com base em profissionais de saúde	4
Estudo descritivo de prevalência com base em serviços de saúde	1
Estudo ecológico	3
Estudo ecológico de séries temporais	6
Estudo ecológico transversal	1
Estudo longitudinal com base em serviços de saúde	1
Estudo longitudinal com base em usuários de serviços de saúde	1
Estudo qualitativo	35
Estudo quanti-qualitativo	12
Estudo transversal	1
Estudo transversal com base em profissionais de saúde	1
Estudo transversal com base em profissionais dos serviços de saúde	3
Estudo transversal com base em serviços de saúde	6
Estudo transversal com base em usuários de serviços de saúde	7
Total geral	90

**Tabela 4:** Distribuição dos artigos selecionados por subdimensão de avaliação de desempenho dos serviços de saúde

Subdimensão de avaliação	n
Acessibilidade	13
Continuidade	44
Efetividade	33
Total geral	90

Para seleção dos trabalhos foram considerados como critérios de inclusão: a) artigos originais em português, espanhol e inglês, publicados em periódicos indexados; b) trabalhos publicados no período entre 2004 a 2018, pois a PNSB, sob o nome Brasil Sorridente, foi implantada em 2004, e por isso entende-se que o estudo sobre a evolução da política deva ser feito no período acima estabelecido. Só foram considerados artigos com texto completo disponível.

### Abordagem Metodológica

Optou-se por utilizar o modelo de avaliação proposto no Projeto de Avaliações de Desempenho de Sistemas de Saúde (PROADESS), se constituiuma das propostas mais abrangentes e estruturadas para avaliação do desempenho de sistemas de saúde, com uma matriz que incorpora dimensões de importância crítica para a avaliação Sistema Único de Saúde. A Matriz Conceitual do PROADESS^6^ traz quatro grandes dimensões para a avaliação do Sistema de Saúde: determinantes da saúde, condições de saúde da população, sistema de saúde e desempenho dos serviços de saúde, sendo que o eixo equidade corta transversalmente todas essas dimensões e que o desempenho deve incluir as subdimensões acesso, efetividade, eficácia, adequação, continuidade, segurança, aceitabilidade e direitos dos pacientes.

## RESULTADOS

Os critérios estabelecidos resultaram em 600 publicações completas (586 na primeira busca e 14 na segunda). Foram excluídos 539 artigos: 151 artigos após análise de duplicidade, 236 artigos após a leitura do título, 45 pelo tipo de publicação e 107 artigos após leitura do resumo, por não se enquadrarem na temática da pesquisa. Ao final, foram selecionadas e analisadas 61 publicações originais e mais 29 publicações em *snowball sampling*, totalizando 90 publicações.

A seleção dos artigos foi realizada por 2 revisores distintos (R1 e R2) que, a partir da chave de busca construída, examinaram individualmente os artigos, seguindo os mesmos critérios de elegibilidade, como a leitura do título, o tipo de publicação e a leitura do resumo para posterior definição sobre sua pertinência ao escopo desse estudo, conforme demonstrado no Fluxograma 1. Depois, os revisores aplicaram critérios de elegibilidade isoladamente na leitura integral dos artigos selecionados, cruzando informações para certificar a integridade dos conteúdos, levando em consideração um conjunto de critérios, como, ano de publicação, delineamento do estudo, características da amostra (tamanho da amostra, local do estudo e período considerado no estudo), entre outros. Em caso de dúvida sobre juízo de inclusão, um terceiro revisor (R3) foi consultado, quando houve divergências, buscando-se debater até se chegar a uma concordância para inclusão ou exclusão do artigo.

Para análise dos artigos e melhor compreensão sobre a configuração da implantação e da implementação da Rede de cuidado em saúde, os estudos foram categorizados em eixos temáticos: 1) acesso, 2) continuidade e 3) efetividade. Nesse processo, observou-se uma lacuna importante, pois não foram encontradas produções classificadas nas outras cinco dimensões consideradas pela matriz conceitual do PROADESS para avaliação de desempenho (segurança, adequação aceitabilidade, eficiência e respeito ao direito).

Os conceitos basilares de acesso, continuidade e efetividade podem ser definidos, respectivamente, como a capacidade do usuário de utilizar os serviços e assistências de saúde no momento adequado, a competência da rede de saúde de proporcionar cuidados de forma continuada e ordenada e, por último, o alcance do melhor resultado possível no cuidado em saúde do paciente.

## DISCUSSÃO

### Acesso

Diversos estudos nacionais^7^ debruçaram-se sobre a oferta de serviços de saúde bucal a partir do acesso, suas desigualdades e da forma de uso em uma rede assistencial. No trabalho de Peres^8^, sobre a utilização de serviços no SUS, a procura por assistência em saúde bucal constituiu a terceira maior demanda.

No campo da atenção básica, a PNSB inequivocamente se constituiu como uma importante ferramenta de continuidade, norteada em princípios fundamentais de integralidade, de universalidade e de equidade, em que a existência de um desses princípios não pode ser entendida sem a inclusão dos outros dois. Para a consecução desses princípios é necessário o cumprimento de etapas de planejamento local, incluindo diagnóstico situacional, priorização de demanda, programação para execução das ações, execução da assistência, acompanhamento e avaliação. Porém, todo ordenamento legal da Política Nacional de Saúde Bucal passa ao largo da organização da oferta de serviços e da coordenação da rede de assistência, voltado majoritariamente para instalação de equipamentos públicos de saúde bucal. Os municípios têm experimentado as mais diversas formas de organização de oferta de serviços de saúde bucal na atenção básica, na medida em que o acolhimento aos usuários se dá por livre demanda, por ações programadas, por atendimentos de urgência ou, ainda, por combinações destes, sem que haja um padrão mínimo de qualidade definido^9^ ou presença de indicadores de acesso em saúde que tenham sido objeto do planejamento loco-regional para conformação da Rede.

Os estudos^10,11^ identificaram sobrecarga no atendimento de urgência de baixa complexidade clínica em unidades de pronto atendimento, que poderiam ser atendidos na atenção básica, mas acabaram transformando o pronto atendimento na porta de acesso fundamental do sistema de saúde, desorganizando a Rede. Em estudo nacional^8^, observou-se que, entre os anos de 1998 e 2008, a grande ampliação no acesso dos serviços de saúde bucal foi evidenciada entre jovens de 7 a 19 anos, reportando assim ao antigo modelo de serviços odontológicos voltado a escolares. Figueiredo e Góes^12^ revelaram importantes desigualdades de acesso aos serviços de saúde bucal, mas reconhecem o aumento de cobertura pela redução do número de pessoas que nunca foram ao dentista. Silva^13^ mostrou que altas coberturas de equipes de Saúde Bucal são facilmente encontradas em municípios com pequeno porte populacional, onde com pequeno número de equipes se alcança mais facilmente coberturas próximas a 100% da população. Contudo ainda permanecem modelos inadequados de oferta, cobertura e gasto público em saúde^14^, já que o modelo dá ao gestor local a liberdade de organização de redes municipais e não torna clara a organização e o financiamento de redes regionalizadas de atenção à saúde e nem aponta para a figura comum em países com sistemas universais de saúde de uma autoridade sanitária regional que organizaria essa Rede.

Casotti^15^, em estudo sobre os resultados do primeiro ciclo do PMAQ-AB, apontou que a simples marcação de consulta não é acessível a 34,5% dos usuários entrevistados nas mais de 12 equipes de saúde bucal avaliadas. O trabalho identificou ainda que 33% dos usuários que buscaram o atendimento foram atendidos no mesmo dia (demanda espontânea e/ou casos de urgência) e que 45,8% dos pacientes foram agendados no prazo de 2 a 15 dias após a marcação (demanda programada). Houve expressivas diferenças macrorregionais, sendo que na Região Norte 48,5% foram atendidos no mesmo dia ou no dia seguinte e 39,7% são agendados no prazo de 2 a 15 após a marcação; e na Região Sudeste 19,2% foram atendidos no mesmo dia ou no dia seguinte, 19,1% entre 16 a 30 após a marcação e 14% esperam mais de 30 dias após a marcação.

Maior cobertura de atenção básica e maior acesso aos serviços ou resolutividade não estão necessariamente relacionados como já demonstraram diversos estudos^16,17^, pois a abertura de um serviço de saúde deve ser orientada pela necessidade epidemiológica do território, obedecendo a um planejamento balizado por um conjunto de indicadores que monitorem e avaliem o desempenho da rede de saúde^7^, em outras palavras, o aumento do acesso possibilitou por um tempo determinado a melhora dos indicadores de saúde da população? Ou ainda, a abertura de um serviço possibilitou que aqueles que mais precisam desse serviço conseguissem acessá-lo em tempo hábil? Outros trabalhos^8,10^ assinalam uma escassez de estudos sobre o monitoramento de indicadores e a avaliação da qualidade dos serviços prestados, em que, por exemplo, Peres^8^ aponta um desequilíbrio entre a utilização de serviços de saúde e os grupos sociais, com evidente prejuízo aos economicamente mais frágeis.

Em estudo realizado^9^ na região metropolitana de Curitiba, verificou-se que, na Rede de Atenção à Saúde Bucal, o acesso é insuficiente e desigual, de acordo com a autora, decorrente da pouca organização no planejamento dessa linha de cuidado e de intervenções assistenciais com forte marca ainda de antigos programas de saúde escolar.

### Efetividade

A efetividade se define como o alcance de objetivos traçados, ou seja, a melhora de indicadores de saúde bucal e a melhora da condição de saúde bucal da população no interior da rede de atendimento nos níveis de atenção, buscando a integralidade. Em outra perspectiva, a população espera ter suas demandas em saúde bucal resolvidas pelo serviço^18^, sendo que a busca pela efetividade passa pela integração dos serviços em redes de assistência, que devem objetivar a eficiência e a racionalidade dos serviços, produzindo economia, expansão da assistência, melhoria do acesso e evitar a duplicidade de serviço^13^.

Casotti^15^, analisando os dados do primeiro ciclo do PMAQ, constataram que quase metade das equipes de saúde bucal no Brasil não possui qualquer protocolo orientando a gestão da clínica ou a organização dos seus processos de trabalho, o que indica pouca racionalidade na rotina de assistência à saúde pelas equipes. Resultados diferentes foram encontrados nos Centros de Especialidades Odontológicas (CEO), em que 78,2% dos odontólogos relataram existir fluxos definidos de referência e contrarreferência em suas rotinas de trabalho. No mesmo estudo, os dados do primeiro ciclo do PMAQ apontam que 85,1% das equipes de saúde bucal fazem acolhimento da demanda espontânea, mas somente 43,8% utilizam algum protocolo para acolhimento, o que corrobora com os outros resultados encontrados em diversos estudos^18^.

Quando analisamos o tempo de espera para consulta de referência nos CEO, percebemos que a estomatologia é a especialidade que apresenta o maior tempo de espera, com 48,3% de todos os pacientes aguardando mais de 1 ano para uma consulta, chegando a 63,8% na Região Norte e 56,5% na Região Nordeste, indicando a fragilidade de uma rede despreparada e com sem resolutividade para acolher esse paciente com suspeita de câncer.

Estudos sobre os CEO realizados nacionalmente e, especificamente, na Região Sul do Brasil^21,22^ verificaram que a maior parte dos CEO não cumprem as metas mensais de produção, estipuladas na Portaria específica para o recebimento de recursos federais de custeio. A maior parte das metas atingidas foram as de procedimentos básicos, fazendo com que o serviço de média complexidade compita com o serviço de atenção básica, corroborando com o argumento anterior, levantado por Chaves^19^, Soares^23^ e Tomassi^24^, e colocando a média complexidade, na prática, como outra porta de entrada do sistema e configurando uma rede com dupla porta de entrada. Ainda, segundo Moura^22^, esse resultado também pode indicar baixa resolutividade da atenção básica, perspectiva corroborada por Barbosa^25^, que analisou o perfil dos encaminhamentos para média complexidade por meio do sistema de regulação, em que uma gama considerável de encaminhamentos para outros pontos de atenção da rede eram procedimentos de baixa complexidade tecnológica, que poderiam ser efetuados na atenção básica/primária, sem a necessidade de deslocamento físico do usuário do SUS, demora na resolutividade dos casos e outros transtornos decorrentes do agravamento dos padrões de doença em virtude da espera.

### Continuidade

Em uma rede de cuidados, os diversos pontos de atenção devem estar interligados, formando vasos comunicantes onde uma alteração em um ponto, direta ou indiretamente, interfira nos demais. Assim, quando há alteração significativa em algum indicador de saúde em um ponto, os demais também repercutem essa mudança de padrão. Diversos estudos apontam a existência serviços de saúde isolados, sem maiores interfaces com demais pontos assistenciais, como apontou Scarparo^20^, que mesmo com o aumento no indicador de primeira consulta odontológica programática e exodontias na atenção básica, a rede analisada coexiste com indicadores de produção de endodontia e periodontia (atenção secundária) constantes no período analisado, ou seja, aumentou o número de pessoas atendidas na atenção básica, mas não houve um acompanhamento da atenção secundária, comportamento similar ao encontrado em Cuiabá^26^ e no Rio Grande do Norte^27^, onde os indicadores odontológicos se comportavam de forma irregular e sem obedecer relações claras de casualidade.

Como a maioria dos municípios brasileiros é de pequeno porte, não se justifica uma rede de média complexidade odontológica que só pode oferecer serviços de atenção básica^28^, essa realidade rompe a lógica de tratamento continuado dentro de um mesmo município, consequentemente exigindo pactuações intermunicipais e estaduais. Assim, a continuidade do cuidado e a integralidade da assistência ofertada pelo SUS dependem de acordos multilaterais entre diversos atores sociais e políticos, o que dificulta a sua execução.

Em um estudo^20^ englobando todos os municípios do Estado do Rio de Janeiro, entre 2004 e 2010, verificou-se um aumento de primeira consulta odontológica programática nos municípios de pequeno e grande porte, enquanto nos municípios de médio porte houve uma retração. Percebeu-se também ampliação em todas as cidades dos procedimentos de atenção básica por habitante, levando ao aumento da continuidade do tratamento. Porém, em seu estudo, Mattos^17^notou a sobreposição da oferta de algumas ações, carência de outras e precária integração entre os serviços, ou seja, a continuidade do cuidado, pilar da integralidade da assistência, em que o usuário deveria ter de forma organizada um conjunto de serviços disponíveis, é afetada pela expansão de equipamentos públicos sem planejamento, o que interfere diretamente na evolução do seu grau de morbidade com impacto no desfecho.

Estudo^28^ sobre o sistema de referência e contra referência na região metropolitana do Rio de Janeiro, observou baixa governança das gestões locais sobre a oferta de vagas da atenção básica para média complexidade, criando um perigoso funil e longa fila de espera, que gera angústia aos usuários e torna mais complexa a expectativa da integralidade do cuidado. Nesse estudo, os fatores que influenciam a formação de longas filas de espera, para especialistas, são semelhantes aos observados em outros estudos, como precário mecanismo de regulação de vagas e limitada oferta de serviços de média complexidade. Supõe-se que as ofertadas na média complexidade, com especialistas, sejam disponibilizadas sem se calcular a real necessidade de absorção desses pacientes da atenção básica, como se a necessidade de tratamentos especializados tivesse que se adequar ao número de vagas disponibilizado e não o contrário. Se compararmos ao dito popular, a oferta de vagas na média complexidade é um alfaiate que, ao notar que o terno ficou menor que o freguês decide alterar o freguês e não o terno.

Outro componente de iniquidade são os municípios com melhores condições, que têm maiores possibilidades de oferta de atendimento integral. Dentro desses mesmos municípios, há grupos específicos com melhores condições de saúde, tornando-se grupos com mais acesso aos serviços. Nesse sentido, as filas de espera são sintomas de uma oferta insuficiente para absorver determinada demanda, tornando o tamanho da fila de espera por serviços de saúde inversamente proporcional à sua necessidade, ou seja, fila de espera longa (grande) deduz rede insuficiente (pequena), tornando a própria rede ineficaz.

Diversos autores^13,15,16,22^ ressaltam que no conjunto das demais políticas de saúde há uma expansão da oferta de serviços sem planejamento e sem programação das ações. Mattos^17^ pondera que a vertiginosa expansão dos serviços de saúde bucal na atenção básica não acompanhou a tímida expansão da rede de média complexidade, não garantindo, assim, a integralidade do cuidado aos usuários.

Debruçando-se sobre a ocupação espacial dos CEO nas cinco macrorregiões brasileiras, Góes^21^ demonstrou que a maior parte dos CEO estava concentrada na Região Nordeste e depois Sudeste, levando a crer que o princípio da equidade estava sendo levado à cabo, dada a grande fragilidade socioeconômica da região. Porém, nota-se também no mesmo estudo que a Região Norte, também frágil socioeconomicamente, apresenta a menor concentração de CEO, mesmo não sendo a região menos habitada, o que sugere que a equidade não foi um eixo central na distribuição desses equipamentos.

Em outro estudo de Góes^21^, observou-se que os CEO implantados estavam alocados em municípios mais populosos, com maiores volumes de receitas municipais para investimento, melhor Índice de Desenvolvimento Humano (IDH) e, consequentemente, com melhores condições de saúde bucal, resultados corroborados por outra pesquisa^30^.

Portanto, a análise dos obstáculos existentes e as limitações apresentadas para o cumprimento de seu objetivo não devem obscurecer os avanços alcançados. Protocolos clínicos, sistemas de referência e contrarreferência, carteiras de oferta de serviços e regulamentação de determinadas ações trazem maior racionalidade para uma rede de saúde. Salienta-se ainda que, apesar do grande percentual de municípios que implantaram as equipes de saúde bucal após a implantação da política, observou-se neste estudo ainda uma baixa cobertura populacional, evidenciando que o número de equipes implantadas nos municípios ainda é diminuto, necessitando ser aumentado para que atendam à maior parte da população. Autores como Góes apontam para necessidade da revisão do cardápio de serviços de especialidades pelos CEO, onde as maiores demandas por serviços, como endodontia, sejam ampliadas e especialidades com menores demandas em saúde bucal sejam inseridas na perspectiva do planejamento loco-regional, respeitando a especificidade de determinadas regiões de saúde.

Logo, mesmo com o aumento significativo e a diversificação da rede assistencial, diversos sinais apontam que a organização dos serviços e sua forma de gerenciamento engessam a formação de redes regionalizadas de atendimento integral à saúde. Destaca-se também que, embora o aumento no número de unidades de saúde e de profissionais na assistência tenha impacto na utilização do serviço ofertado, não há mecanismos institucionalizados de mensuração da qualidade do serviço prestado nem fortes contratualizações de metas e indicadores, exceção aos três ciclos do PMAQ-AB.

Observando a Matriz Conceitual do PROADESS e suas oito subdimensões, outro ponto importante que os artigos selecionados trouxeram foi o número baixo de estudos que abordem temáticas distintas de acesso, efetividade e continuidade, ou seja, o volume de publicações encontradas sobre o objetivo do estudo tinha pouca diversidade temática. Assim, torna-se necessária a avaliação da Rede de Saúde Bucal de forma mais abrangente e em seus mais variados aspectos.

Apesar dos esforços na busca pela maior quantidade possível de trabalhos sobre a temática abordada, uma importante limitação do estudo é a própria carência de artigos publicados nas fontes pesquisadas. Apesar de serem poucos artigos sobre o tema e suas variáveis isso não desqualifica os resultados encontrados dada a relevância dos trabalhos selecionados, haja vista que também não buscamos esgotar o assunto, mas somá-lo na perspectiva da melhor compreensão sobre a política implantada.
